# Stiffness Prediction of Connections between CHS Tubes and Externally Welded I-Beams: FE Analyses and Analytical Study

**DOI:** 10.3390/ma13133030

**Published:** 2020-07-07

**Authors:** Sabatino Di Benedetto, Massimo Latour, Gianvittorio Rizzano

**Affiliations:** Department of Civil Engineering, University of Salerno, via Giovanni Paolo II 132, 84084 Fisciano (SA), Italy; mlatour@unisa.it (M.L.); g.rizzano@unisa.it (G.R.)

**Keywords:** stiffness, FEM, experimental, parametric analysis, regression analysis, component method

## Abstract

Double-tee profiles are the most popular members in Europe and the USA for steel structures. However, more efficient cross-sections, such as circular hollow sections (CHSs), could be adopted, since they can provide higher aesthetic, economic and mechanical benefits, with the only drawback of more complex connections such as in the case of I-beams welded to the external surface of circular hollow profiles. Based on the ring model theory, developed by Togo, a rule to design the flexural resistance of such a connection has been included in the Eurocode 3 part 1.8, while no formulations are provided to predict the corresponding initial stiffness. The present work aims at filling this knowledge gap, adopting an approach based on experimental, numerical and analytical work. A monotonic and a cyclic test have been performed on two beam-to-column sub-assemblies; the experimental outcomes have been exploited to validate a finite element (FE) model developed in Abaqus and used to numerically perform the monotonic loading simulations of 30 joints. Afterwards, starting from the extracted information about stiffness, a regression analysis was carried out to define the coefficient of a design equation analytically derived applying the component method approach. The regression analysis is characterized by a coefficient of variation equal to 0.19.

## 1. Introduction

The use of double-tee profiles for the structural members of steel moment-resisting frames (MRFs) is widespread in Europe and the USA. This large use is due to the existence of reliable formulations able to predict, in a very accurate way, the mechanical behaviour of I- or H-shape members, but also to the possibility to adopt a wide range of easy-to-construct beam-to-column connections [1,2,3,4,5,6,7,8,9,10,11,12,13,14,15,16,17,18,19,20,21,22,23]. Additionally, for most of the existing beam-to-column connection solutions, the flexural strength and stiffness can be accurately predicted by applying the component method [24,25,26,27]. The reliability of such a modelling tool has been proven by research works dealing with the study of partial-strength joints, whose weak elements consist in the panel zone [3,28,29], the end-plate [3,28,30], or the T-stubs [4,7,9].

Nevertheless, while in Europe and the USA, the use of I- or H- shapes is more common, in Japan, due to the benefits deriving from the equal inertia, the use of a hollow section is more widespread. This difference depends mainly on a technical reason. In fact, while in Europe and the USA, perimetral frames are more common, in Japan, space frames are more widely used. In space frames, the symmetry of the tubular sections allows for the better exploitation of the characteristics of these profiles which, in this particular scheme, are mainly subjected to biaxial bending. In many cases, due to the circular-symmetry, circular hollow section (CHS), members are preferred in the design practice due to the following features: (i) the high values of the radius of gyration, due to the ideal material distribution; (ii) the absence of a weak axis, which characterizes, instead, the double-tee profiles; (iii) the low surface area, compared to the double-tee profiles, with the benefit of reducing paintings, fire and corrosion protection costs; (iv) the lower drag coefficients affecting wind forces; (v) the possibility of reducing the cost of transportation and assembly of the members; (vi) the higher aesthetical aspect.

This cross-section typology has been investigated, starting from 1924 when Greene [31] experimentally studied the axial compression capacity of CHS profiles. Afterwards, these efforts were carried on by Bouwkamp [32], Marzullo and Ostapenko [33], Chen and Ross [34], Elchalakani, Zhao and Grzebieta [35], Ma, Chan and Young [36], Xiong, Xiong and Liew [37], Meng and Gardner [38], as is well reported by Meng et al. [39] Instead, the mechanical flexural behaviour was studied by Korol and Hubonda [40], Willhoit Jr. and Mervin [41] and Sherman [42], while the combined compression and bending action has been investigated by Prion and Birkemoe [43], O’Shea and Bridge [44], Pan [45], Nseir [46], Ma [47], Pournara et al. [48] and Meng and Gardner [38].

Even though the above-mentioned works confirm the huge efforts in studying CHSs, nevertheless, their application has been mainly limited to bracing systems and truss structures, because of their high axial resistance. Recently, instead, many applications are being carried out, exploiting CHSs with concrete as composite members in MRFs [37,49], losing, in such a way, the easy-to-construct benefit induced by steel members, because of the required pouring concrete on site. 

Another reason which has limited the use of CHS profiles as column members is related to the high complexity of the beam-to-column connections. Regarding this topic, different solutions have been investigated, as has already been reported in [50,51]. For the sake of clarity, the most adopted details are characterized by: (i) external welded I-beams [52,53,54,55]; (ii) columns which are interrupted in order to insert plates connected to the beam flanges [56]; (iii) collar plates able to ensure the connection between the beam flanges and the column [57,58,59,60]; (iv) composite columns [61,62,63,64].

The main advantage of the fast constructional process ensured by the first solution is invalidated by the low stiffness and resistance the connection is able to provide, making it suitable only in the case of pinned or semi-continuous frames [65]. Nevertheless, a formulation to predict the flexural strength of such a kind of joint has been derived based on the ring model theory developed by Togo [66] and subsequently deepened by Wardenier, de Winkel, Packer and Zhao [67,68,69,70,71,72]. Referring to the second and the third solution, instead, complex structural details have to be conceived, but with the benefit of increasing both stiffness and resistance [73,74]. Instead, the last solution is out of interest because of the required pouring concrete on-site, which slows the construction process [54,75].

Another recently studied alternative consists of conceiving a connection with a beam which passes through the tubular column. Such a solution has only been recently introduced thanks to the adoption of 3D-laser cutting technology (3D-LCT) in the field of civil engineering. However, many efforts have been devoted to this topic, as it is clearly referring to the works by Voth [65,75] and Kanyilmaz [73,74].

Within this framework, at the STRENGTH (STRuctural ENGineering Test Hall) laboratory of the University of Salerno, an experimental campaign has been carried out, with the aim of studying both the flexural yielding strength and stiffness of connections between CHS tubes and externally welded or through-all IPE profiles. In fact, two preliminary works have regarded the study of the flexural strength [50] and the initial stiffness [51] of the connection with the through I-beam; nevertheless, since there is still a lack of knowledge about the assessment of the initial stiffness of CHS to externally welded I-beam connections, this paper deals with the study of such a topic, with the purpose of proposing a formulation theoretically derived, applying the component method approach. The novelty carried out by the present work consists in allowing designers to take into account the real stiffness of the analysed beam-to-column connection in their numerical models, since no design equations are proposed in the current Eurocode 3 part 1.8 [55].

The research activity has included experimental, numerical and theoretical phases. The experimental campaign consisted of testing, through a cyclic and a monotonic loading history, respectively, two similar connections characterized by CHS columns and externally welded I-beams, with the same geometric and material properties. Afterwards, a numerical model of the tested connections, developed thanks to the finite element (FE) software Abaqus [76], was validated against the experimental results. This has been very useful to numerically model 30 different joints obtained by varying, in a wide range, the main geometrical properties of the connected profiles. The 30 FE models have been submitted to static analyses, performing monotonic simulations to obtain the values of the initial stiffness for each of the cases. These results have been exploited to perform a regression analysis, to calibrate a coefficient belonging to the design equation theoretically derived by applying the component method approach.

## 2. Experimental Activity

### 2.1. Tested Specimens and Experimental Set-Up

**Figure 1:**
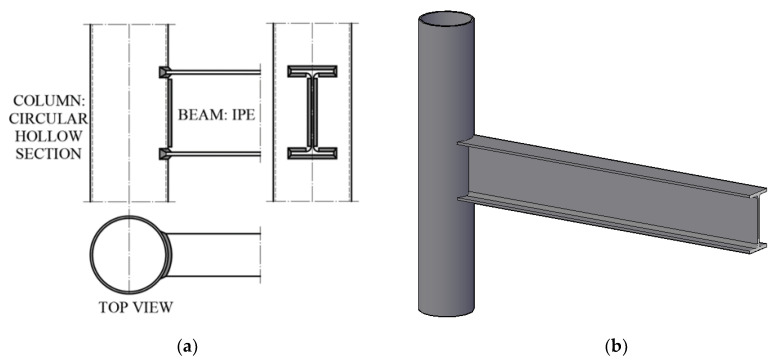
Analysed connection: plan-views (**a**) and 3D view (**b**).

A beam-to-column sub-assembly has been properly designed, in order to manufacture two specimens characterized both by the same geometric and mechanical properties. In particular, referring to the geometry, circular hollow section columns with diameter, thickness and length equal to 219.1 mm, 6 mm and 2000 mm, respectively, and IPE240 beams with a length equal to 1700 mm have been selected (Figure 1). Instead, concerning the mechanical material properties, S355 steel grade has been chosen. Since this study relies on the need of investigating the yielding bending moment and stiffness of the analysed joint typology, the choice of the above members has been inspired by the need of obtaining a local plasticization of the CHS at the flange-to-tubular attachments.

**Figure 2:**
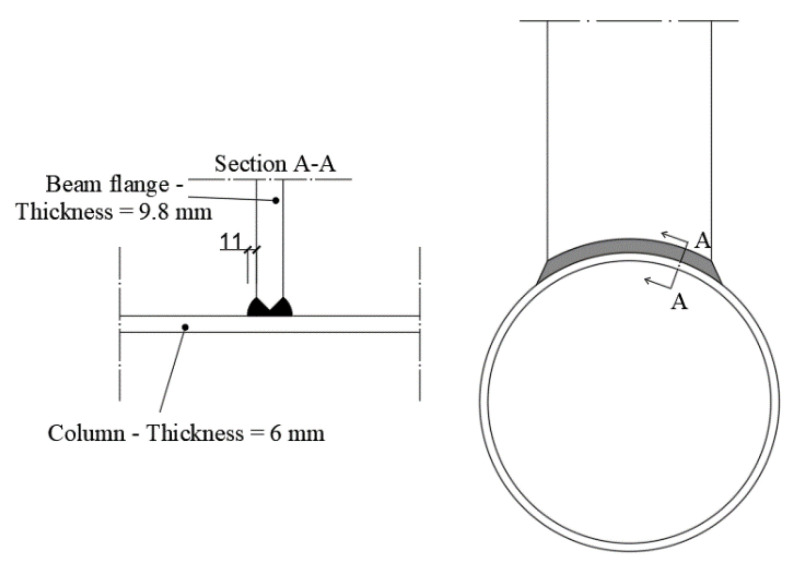
Welding detail.

The connection among the beam and the column has been ensured, externally welding the double-tee profile to the CHS; to this scope, the IPE240 has been cut, on the side connected to the beam, according to the shape of the tubular section, to allow manufacturing full penetration welds with an additional external fillet weld, with leg throat equal to 11 mm (Figure 2).

**Figure 3:**
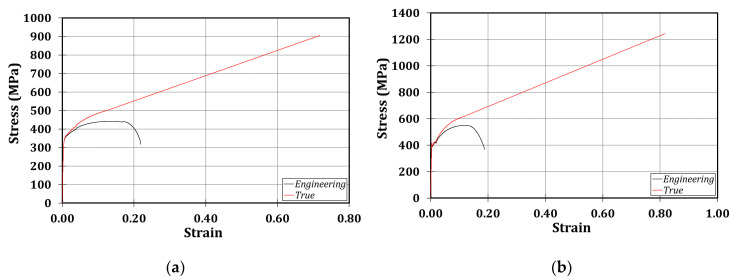
Results of the coupon tests: stress-strain laws referred to the column, f_y_ = 348.2 MPa (**a**); stress-strain laws referred to the beam, f_y_ = 391.9 MPa (**b**).

The mechanical material properties of the tested members have been defined thanks to tension coupon tests extracted from the specimen submitted to the monotonic loading and processed according to the rules provided by Eurocode 3 part 1.4 [77] provision. These tests have allowed us to establish that the chosen profiles were made of S355JR steel grade, as is clear also in Figure 3.

**Figure 4:**
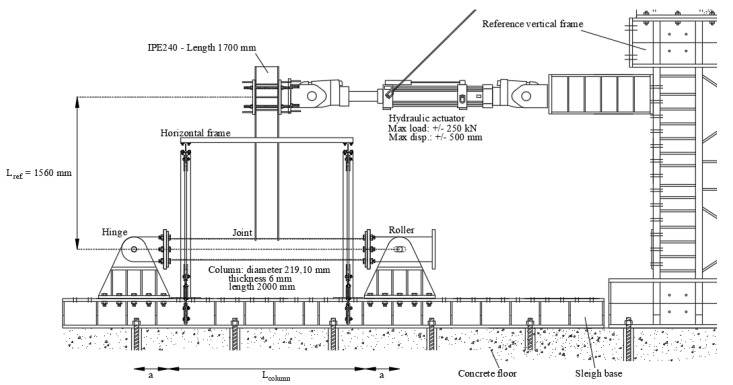
Schematic drawing of the experimental set-up.

Referring to the conceived beam-to-column sub-assembly, two specimens have been manufactured, in order to perform a monotonic and a cyclic test, adopting the same testing rig. In particular, to easily apply a displacement history at the free end of the beam, the experimental set-up has been conceived, placing the column horizontally and restraining its ends with a roller and a hinge to a steel rigid basement. Consequently, the beam, placed in the vertical position, has been connected, at its free end, to a horizontal actuator MTS 243. The other end of the actuator has been connected to a rigid wall made of a steel frame (Figure 4). The actuator has a load capacity of ±250 kN, and a piston stroke of ±500 mm. Additionally, in order to avoid the undesired lateral-torsional buckling phenomena of the beam, an additional horizontal frame has been applied, to constrain the beam section located at 1380 mm from the centre of the tubular section.

The monotonic test has been performed, applying an increasing displacement at a rate of 4 mm/min, while the cyclic test has been performed applying a displacement history at increasing amplitudes according to AISC 341-16 [78].

As it has already been highlighted in [50,51], the bending moments (M) and the rotations (φ) have been assessed thanks to Equations (1) and (2), where $d_{actuator}$ is the displacement applied by the actuator, $F_{actuator}$ is the corresponding recorded force and $L_{ref.}$ is a geometric parameter representative of the distance between the column centerline and the actuator axis.

(1) $$M=F_{actuator}L_{ref.}$$

(2) $$\varphi =\frac{d_{actuator}}{L_{ref.}}$$

**Figure 5:**
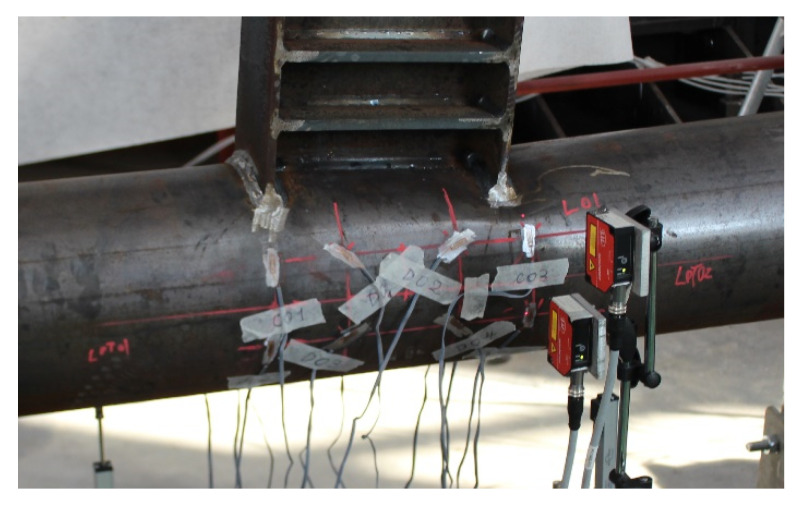
Arrangement of strain-gauges and transducers.

The monotonically loaded specimen has been equipped with 16 strain-gauges applied on the external surface of the CHS in the panel zone, and two LDTs (linear displacement transducers) on the back side of the tubular profile, sufficiently far from the panel zone. The pattern of the strain-gauges has been conceived as reported in Figure 5, to study the distribution of plastic deformations in the panel zone, and the circumferential strips located at the level of the beam flanges.

### 2.2. Experimental Results

**Figure 6:**
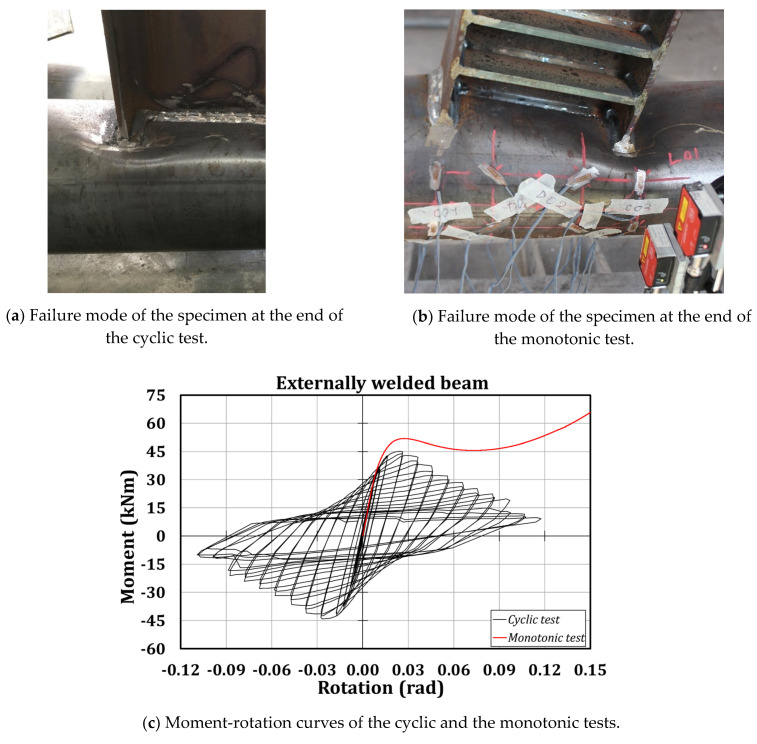
Tested specimens subjected to cyclic and monotonic loading histories.

In this section, the experimental results are discussed. It is worth highlighting that the chord failure crisis of the two specimens occurred without the development of plastic deformations in the beams (Figure 6). As expected, the failure consisted in the local plasticization of those parts of the tubular section close to the flange-to-column attachments, without any damage in the welds. Such a behaviour is the consequence of the high deformability exhibited by the analysed connection when experiencing high displacements at the flanges of the beam inducing a local transverse crushing of the tube. Referring to the monotonic test, it has been possible to observe an initial stiffness equal to 4151 kNm/mrad and a resistance of about 52 kNm; the cyclic test has confirmed the mechanical behaviour of the connection monitored in the monotonic test, since the stiffness is equal to 3849 kNm/mrad, and the resistance is 45 kNm, not far from the values predicted adopting the design formulation proposed by the EC3 part 1.8 [55], and equal to about 37 kNm.

Based on the evidence that the resistance of the analysed joint is at least equal to 45 kNm, which is lower than the flexural strengths of the column (74 kNm) and the beam (124 kNm), and according to both Eurocode 8 part 1.1 [79] and Eurocode 3 part 1.8 [55], the analysed connection can be classified as a partial-strength joint.

**Figure 7:**
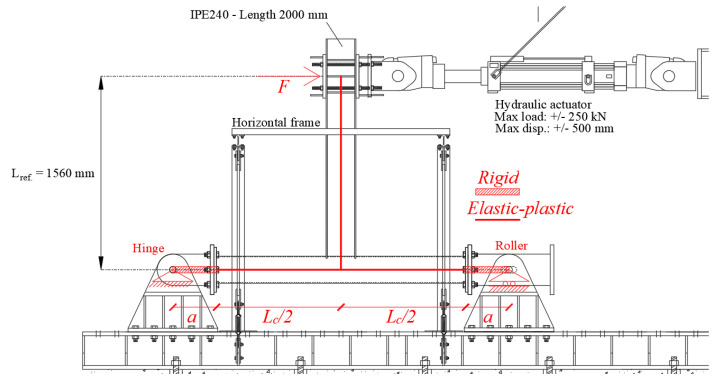
Structural scheme referring to which Equation (3) has been derived (adapted from [51]).

Instead, to classify the joint also in terms of stiffness, it is necessary to detract, from the elastic initial stiffness, the contributions associated with the elastic deformation of the beam and the column according to Equation (3), and the scheme reported in Figure 7:

(3) $$\varphi =\varphi_{exp.}-\frac{FL_{ref.}^{2}}{3EI_{b}}-\frac{2}{3}\frac{FL_{ref.}^{\;}}{EI_{c}}\frac{{\left(a+\frac{L_{c}}{2}\right)}^{3}-a^{3}}{{\left(L_{c}+2a\right)}^{2}}$$

where F is the force recorded by the actuator after having applied the target displacement, φ is the plastic rotation of the connection, $\varphi_{exp.}$ is the chord rotation, as already reported in Equation (2), $I_{b}$ and $I_{c}$ are the moments of inertia of the IPE and the CHS profiles, respectively, $L_{ref.}^{\;}$ is the vertical distance between the intersection of the steel members and the actuator; $L_{c}$ is the column length and a is representative of the rigid parts at the column constraints.

The outcome of the monotonic test has revealed a plastic initial stiffness of about 7.41 kNm/mrad, which allows one to classify the connection as semi-rigid, complying with the Eurocode 3 part 1.8 [55] and referring to the geometric properties of the specimen.

In Figure 6, it is possible to note additional plates welded to the beam flanges and web of the specimen experiencing the monotonic loading. Such a choice can be justified by the need to prevent the local buckling of the beam flanges expected in the case of high rotations, to force the plastic engagement of the connection only.

## 3. Numerical Simulations

### 3.1. FE Modelling

With the aim of studying a wide range of cases of beam-to-column connections analogous to those described in the previous paragraph, it has been chosen to develop a FE model of the tested specimens with Abaqus software [76], and to validate it against the experimental results.

**Figure 8:**
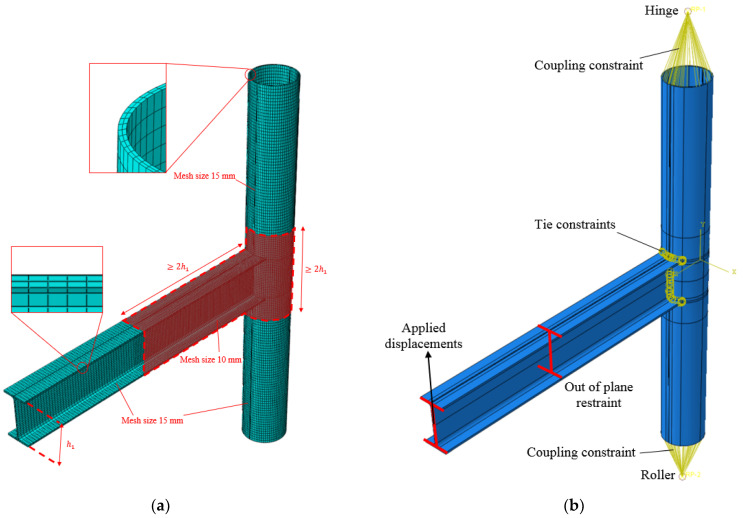
Mesh size selection (**a**) and finite element (FE) model (**b**).

The beam and the column have been geometrically defined by extruding their cross-sections along the longitudinal direction. It is worth specifying that the beam has been properly cut at one of its ends, adopting a cutting circular shape geometrically similar to the external diameter of the tube, in order to allow the connection of the members. In particular, this connection has been ensured without explicitly modelling the welds but assigning tie constraints to the contact zone of the members. To this scope and also to obtain an accurate meshing of the profiles, the members have been properly partitioned. Moreover, a mesh size equal to 10 mm has been applied to the parts of the connection close to the panel zone, while a 15 mm mesh size (Figure 8a) has been assigned to the remaining parts. Nevertheless, it has also been imposed to have at least two elements, embedded both in the thickness of the beam flanges and the column (Figure 8a).

**Figure 9:**
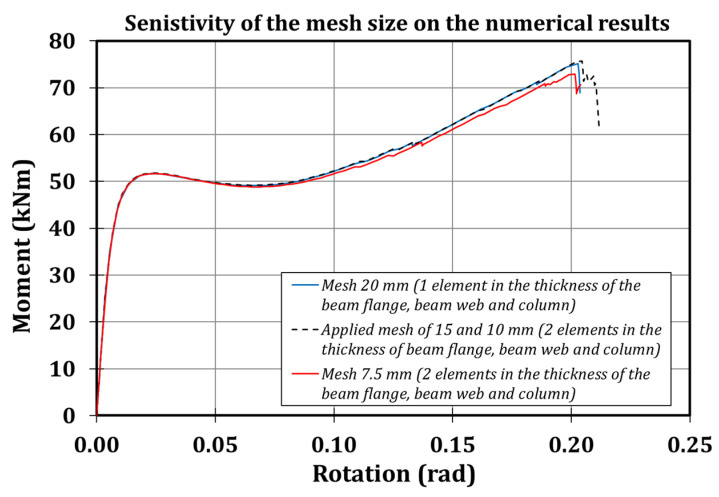
Sensitivity of the mesh size on the numerical results.

The above-mentioned mesh size has been selected, referring to a sensitivity analysis consisting in applying monotonic loading at the free end of the beam of the FE model, and modifying both the mesh size and the number of elements embedded in the thickness of the beam flange, beam web and column (Figure 9). As is clear, the reduction of the mesh size under 10 mm does not significantly affect the results.

An elastic-plastic stress-strain law has been exploited to model the mechanical material properties of both the members, fixing the values of the Young’s modulus and the Poisson’s ratio equal to 210,000 MPa and 0.30, respectively, and exploiting the isotropic hardening to model the plastic branch complying with [28].

The members have been meshed thanks to the 8-node linear brick elements (C3D8-type). Moreover, in order to predict the failure mode, parameters related to the damage evolution have also been properly set, referring to the works by Faralli [80] and Pavlovic et al. [81] for S355 steel grade. These studies have been of primary importance, since they have allowed us to define the equivalent plastic displacement at fracture, ${\bar{u}}_{f}^{pl}$, according to Equation (4):

(4) $${\bar{u}}_{f}^{pl}=\lambda_{S}\lambda_{E}L_{E}\left(\varepsilon_{f}^{pl}-\varepsilon_{n}^{pl}\right)=4.8$$

where $\;\lambda_{S}$ is a parameter equal to 0.928 according to [81]; depending on the adopted minimum value of the mesh size ($L_{E}$), $\lambda_{E}$ is equal to 2.1 in the case of S355JR steel grade, $\varepsilon_{f}^{pl}$ and $\varepsilon_{n}^{pl}$ are the true plastic strains at failure and at the onset of necking, respectively.

The constraints of the specimen have been modelled restraining, with the corresponding degrees of freedom, two reference points located at a distance of 350 mm from the ends of the column, and rigidly connected to them through the coupling tool. Instead, lateral restraints have been applied at the beam section located at 1380 mm from the column face, in order to prevent the beam lateral buckling. The simulations have been performed adopting a static solver and assigning to the free beam-ends the same loading histories experienced by the real-scale specimens (Figure 8b).

Moreover, according to the 80% of maximum fabrication tolerance provided by the Eurocode 3 part 1.5 [82], and to the construction tolerances provided by EN10034 [83], imperfections have been accounted for in the numerical model, amplifying the relevant buckling modes elated to the buckling of the beam flanges and the beam-to-column attachments.

### 3.2. Validation

**Figure 10:**
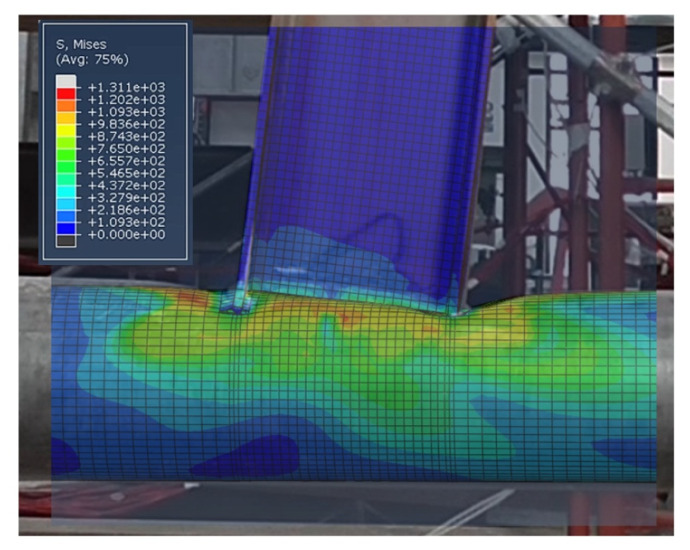
Overlap of FE and experimental results.

**Figure 11:**
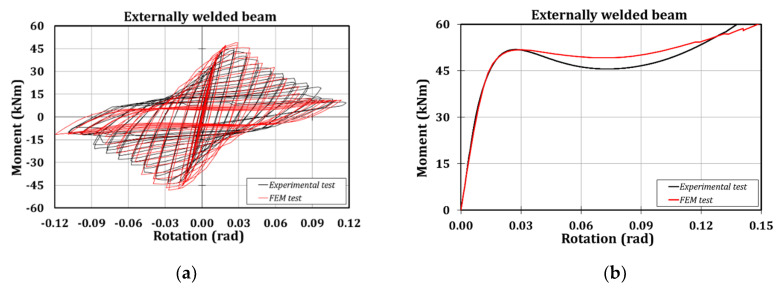
Experimental versus FE results: cyclic test (**a**) and monotonic test (**b**).

The numerical outcomes are consistent with the experimental results, showing a high accuracy, both in terms of prediction of the failure mode (Figure 10) and the moment-rotation hysteretic curve (Figure 11).

Observing Table 1, it is clear that there are negligible scatters between the experimental and the numerical outcomes: 2% in terms of stiffness and 8% in terms of resistance.

### 3.3. Parametric Analysis

Aiming to study a wide range of CHS to externally welded I-beam connections, the validated FE model has been exploited to perform a parametric analysis, performing 30 simulations on the studied joint typology, varying the geometric properties of the members. The selection of the profiles has been ruled by the need of covering a wide range of the main geometric parameters, as already explained in [50,51], namely: $\beta =b_{bf}/d_{0}$, $\gamma =d_{0}/\left(2t_{0}\right)$ and $\eta =h_{b}/d_{0}$ (where $b_{bf}$ is the width of the beam flange, $d_{0}$ is the diameter of the tubular profile, $t_{0}$ is the column thickness and $h_{b}$ is the height of the beam), according to the Eurocode 3 part 1.8 [55]. The range of variation of β is between 0.47 and 0.70, γ between 15.28 and 33.87, and η between 1.02 and 1.55. In Table 2, the geometrical properties of the selected cases are reported. Moreover, it is worth highlighting that, in the hypothesis of having beam-to-column connections characterized by a span/depth ratio equal to 15, it has been assumed to fix the length of the beams equal to 7.5 times the height of the beam.

All the selected cases have been submitted to monotonic tests without applying axial forces to the columns. As outcomes, the obtained moment-rotation curves have allowed one to obtain the initial stiffness for all the analysed cases.

## 4. Proposal of a Design Equation

### 4.1. Theoretical Approach

The present paper aims to define a formulation to predict the initial stiffness of connections between CHS columns and externally welded IPE profiles. In order to reach such a scope, the component method theory has been applied: it consists in preliminary defining the connection components able to provide high sources of deformability, as is done in Eurocode 3 part 1.8 [55], in the case of welded beam-to-column joints. In this case, three components have been identified:

- the hollow section in shear (hss);
- the hollow section in transverse compression (hsc);
- the hollow section in transverse tension (hst).

**Figure 12:**
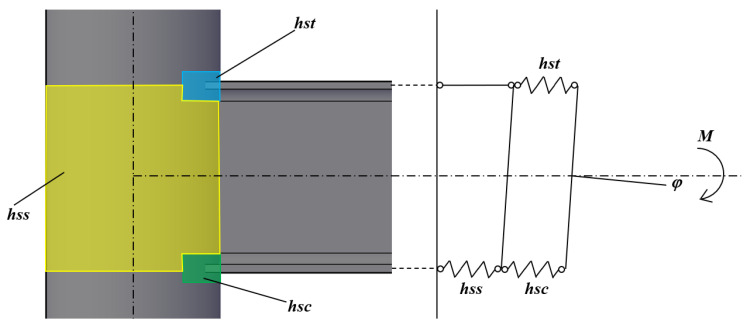
Main sources of deformability: hss (hollow section in shear), hsc (hollow section in transverse compression), hst (hollow section in transverse tension).

In particular, the hsc and hst act as components in parallel, as is clear in Figure 12.

Observing the identified components, it is possible to note that they can be ideally seen as the counterparts of the components belonging to the classical welded joints included in the Eurocode 3 part 1.8 [55].

In particular, for the case of the hollow section in shear (hss), it is possible to refer to the same formulation provided for the column web in shear, in the case of welded connections between double-tee profiles, and reported as Equation (5):

(5) $$k_{s}=\frac{0.38A_{v}}{\beta_{V}z}$$

where 0.38 comes from the ratio $\frac{1}{2\left(1+\nu \right)}$ with ν equal to 0.3, z is representative of the distance between the mid-thickness of the beam flanges, $\beta_{V}=1-h_{b}/\left(L_{c}+2a\right)$ is a reduction factor which accounts for the beneficial effect of the shear in the column, while $A_{v}$ is the shear area of the tubular profile, which is equal to half of the area of the circular hollow section, as reported by Steinboeck et al. [84] Considering that the area of the circular hollow section is equal to $\pi d_{0}t_{0}$, where $d_{0}$ is the diameter of the CHS and $t_{0}$ its thickness, and considering that $z≅h_{b}$ ($h_{b}$ is the height of the beam), the stiffness of this component can be defined as reported by Equation (6):

(6) $$k_{s}=\frac{\pi d_{0}t_{0}}{4\left(1+\nu \right)\beta_{V}h_{b}}$$

Instead, the stiffness of the CHS in transverse compression and tension can be assessed by referring to the same formulation adopted in the case of the column web in tension and compression, as provided by the Eurocode 3 part 1.8 [55], and reported as Equation (7):

(7) $$k_{cwc/cwt}=\frac{0.7b_{eff,c/t}t_{cw}}{d_{c}}$$

Nevertheless, in order to account for the influence that the different geometry of the connection has on its flexural behaviour, the constant 0.7 should be substituted with the coefficient a, which needs to be calibrated against numerical outcomes. The parameters $t_{cw}$ and $d_{c}$, representative of the thickness of the column web and the clear depth of the column web, respectively, are substituted with $t_{0}$ and $d_{0}$. Moreover, $b_{eff,c/t}$ is the effective width in compression or tension and it is usually dependent on the beam flange thickness ($t_{bf}$). The multiplicative coefficient a in Equation (8) covers another important role: it allows one to fictitiously account for the possible influence of other parameters on the effective width.

(8) $$k_{hsc/hst}=\frac{at_{bf}t_{0}}{d_{0}}$$

Therefore, with reference to the mechanical model shown in Figure 12, the stiffness of the joint can be predicted as (Eurocode 3 part 1.8 [55]):

(9) $$k_{j}=\frac{Ez^{2}}{\sum^{\;}\frac{1}{k_{i}}}$$

where $k_{i}$ is the translational stiffness of component *i*.

Substituting Equations (6) and (8) into Equation (9), it is possible to obtain the flexural stiffness reported in Equation (10):

(10) $$k_{\varphi }=\frac{Et_{0}{\left(h_{b}-t_{bf}\right)}^{2}}{\frac{4\left(1+\nu \right)}{\pi }\beta_{V}\eta +\frac{\gamma \xi_{f}}{a}}$$

where $\xi_{f}$ is equal to the ratio between the thickness of the column and the thickness of the beam flange ($t_{0}/t_{bf}$).

### 4.2. Regression Analysis and Assessment of the Accuracy of the Proposed Design Equation

**Figure 13:**
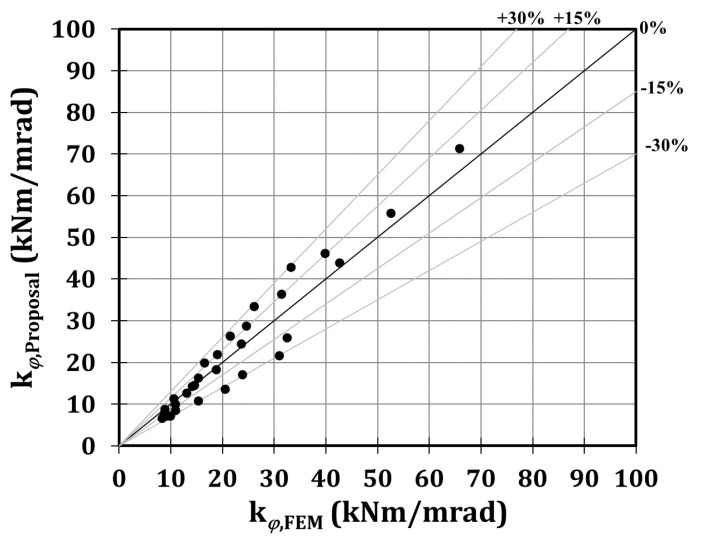
Proposal vs. FEM stiffness.

Starting from the results of the parametric analysis, the coefficient a has been calibrated thanks to a regression analysis, and it is equal to 1.33, inducing a quite accurate prediction of the initial stiffness, since the mean value of the ratios between the stiffness of the proposal and the FE models is equal to 0.98, with a coefficient of variation equal to the 19%, as is reported in Table 3 and Figure 13. In particular, in Figure 13, the results of the 30 cases in terms of the numerical and analytical stiffness are reported. In fact, each point is representative of an analysed case, since its coordinates correspond to the numerical stiffness provided by the FE analyses (*x*-axis) and the corresponding stiffness obtained applying the proposal formulation (*y*-axis). As is clear, the distance of a point from the bisector axis is a parameter to assess the accuracy of the proposed formulation.

## 5. Conclusions

The aim of the present study is to propose a formulation to predict the initial stiffness of connections between circular hollow section columns and double-tee beams externally welded to the tubular profiles. The theoretical background over which the proposed equation relies on is represented by the component method approach, which is supported by both experimental and numerical activities. In fact, two experimental tests have been carried out, and they have been used to calibrate the main parameters of a FE model of the analysed connection typology, which has been useful to perform, employing numerical simulations, a parametric analysis on 30 joints properly selected by varying the main geometrical parameters able to affect the response of the analysed connection typology. Therefore, it has been possible to calibrate the only unknown coefficient of the proposed analytical formulation through a regression analysis.

The most significant result provided by the present work is the proposal of a formulation to predict the initial stiffness of beam-to-column joints between circular hollow section columns and externally welded double-tee beams. The main outcome is that such a formulation is structurally similar to those currently included in the Eurocode 3 part 1.8 [55], referring to connections between I-beams and H-columns.

However, because of the different and more complex geometry of the derived connection, additional parameters have been introduced: $\xi_{f}$, γ and η. The proposal has proven to be accurate, since the mean value of the ratios between the stiffness derived analytically and the stiffness obtained by the numerical simulations is equal to 0.98, with a coefficient of variation equal to 0.19. Moreover, the field of application of the proposed equation has to be limited only to the connections whose geometric parameters β, γ and η belong to the ranges of variability used to select the above mentioned 30 cases.

## Figures and Tables

**Table 1:** Experimental vs. finite element (FE) results: stiffness and resistance information.

	Stiffness (kNm/rad)			Resistance (kNm)		
	Exp.	FEM	FEM/Exp.	Exp.	FEM	FEM/Exp.
**Monotonic test**	4151	4072	0.98	52	52	1.00
**Cyclic test**	3849	3786	0.98	45	49	1.08

**Table 2:** Analysed cases.

Test	Column (Diameter/Thickness)	Beam	*β*	*γ*	*η*	Stiffness (kNm/mrad)
1	193.7/6	IPE240	0.62	16.14	1.24	9.84
2	219.1/4	IPE300	0.68	27.39	1.37	9.20
3	219.1/6	IPE240	0.55	18.26	1.10	8.29
4	219.1/6	IPE270	0.62	18.26	1.23	10.89
5	219.1/6	IPE300	0.68	18.26	1.37	15.30
6	219.1/6	IPE330	0.73	18.26	1.51	20.45
7	244.5/8	IPE330	0.65	15.28	1.35	23.80
8	244.5/8	IPE360	0.70	15.28	1.47	30.89
9	273/5	IPE300	0.55	27.30	1.10	8.67
10	273/5	IPE330	0.59	27.30	1.21	10.86
11	273/5	IPE360	0.62	27.30	1.32	13.06
12	273/8	IPE400	0.66	17.06	1.47	32.41
13	323.9/5	IPE330	0.49	32.39	1.02	8.76
14	323.9/5	IPE360	0.52	32.39	1.11	10.55
15	323.9/5	IPE400	0.56	32.39	1.23	14.57
16	323.9/6.3	IPE360	0.52	25.71	1.11	14.07
17	323.9/6.3	IPE400	0.56	25.71	1.23	18.74
18	323.9/6.3	IPE450	0.59	25.71	1.39	23.57
19	355.6/6	IPE400	0.51	29.63	1.12	15.33
20	355.6/6	IPE450	0.53	29.63	1.27	18.92
21	355.6/6	IPE500	0.56	29.63	1.41	24.62
22	355.6/6	IPE550	0.59	29.63	1.55	31.33
23	355.6/6	IPE600	0.62	29.63	1.69	39.76
24	406.4/6	IPE450	0.47	33.87	1.11	16.45
25	406.4/6	IPE500	0.49	33.87	1.23	21.49
26	406.4/6	IPE550	0.52	33.87	1.35	26.11
27	406.4/6	IPE600	0.54	33.87	1.48	33.18
28	406.4/10	IPE500	0.49	20.32	1.23	42.58
29	406.4/10	IPE550	0.52	20.32	1.35	52.54
30	406.4/10	IPE600	0.54	20.32	1.48	65.79

**Table 3:** Comparison between the stiffness provided by the numerical outcomes and by the proposed formulation.

Test	Column (d_0_/t_0_)	Beam	*k_ϕ__, FEM_* (kNm/mrad)	*k_ϕ__, Proposal_* (kNm/mrad)	Proposal/FEM
1	193.7/6	IPE240	9.84	7.18	0.73
2	219.1/4	IPE300	9.20	7.24	0.79
3	219.1/6	IPE240	8.29	6.64	0.80
4	219.1/6	IPE270	10.89	8.58	0.79
5	219.1/6	IPE300	15.30	10.86	0.71
6	219.1/6	IPE330	20.45	13.67	0.67
7	244.5/8	IPE330	23.80	17.12	0.72
8	244.5/8	IPE360	30.89	21.68	0.70
9	273/5	IPE300	8.67	7.84	0.90
10	273/5	IPE330	10.86	9.97	0.92
11	273/5	IPE360	13.06	12.70	0.97
12	273/8	IPE400	32.41	25.96	0.80
13	323.9/5	IPE330	8.76	8.83	1.01
14	323.9/5	IPE360	10.55	11.33	1.07
15	323.9/5	IPE400	14.57	14.58	1.00
16	323.9/6.3	IPE360	14.07	14.27	1.01
17	323.9/6.3	IPE400	18.74	18.37	0.98
18	323.9/6.3	IPE450	23.57	24.46	1.04
19	355.6/6	IPE400	15.33	16.39	1.07
20	355.6/6	IPE450	18.92	21.91	1.16
21	355.6/6	IPE500	24.62	28.80	1.17
22	355.6/6	IPE550	31.33	36.46	1.16
23	355.6/6	IPE600	39.76	46.20	1.16
24	406.4/6	IPE450	16.45	19.92	1.21
25	406.4/6	IPE500	21.49	26.34	1.23
26	406.4/6	IPE550	26.11	33.54	1.28
27	406.4/6	IPE600	33.18	42.79	1.29
28	406.4/10	IPE500	42.58	43.90	1.03
29	406.4/10	IPE550	52.54	55.90	1.06
30	406.4/10	IPE600	65.79	71.31	1.08
			Mean=		0.98
			Standard deviation=		0.18
			Coefficient of variation=		0.19
